# Full-length transcriptome analysis of shade-induced promotion of tuber production in *Pinellia ternata*

**DOI:** 10.1186/s12870-019-2197-9

**Published:** 2019-12-18

**Authors:** Tao Xue, Han Zhang, Yuanyuan Zhang, Shuqin Wei, Qiujie Chao, Yanfang Zhu, Jingtong Teng, Aimin Zhang, Wei Sheng, Yongbo Duan, Jianping Xue

**Affiliations:** Key Laboratory of Resource Plant Biology of Anhui Province, College of Life Sciences, Huaibei Normal University, Huaibei, 235000 China

**Keywords:** *Pinellia ternata*, Shade, Transcriptome, Single-molecule real-time sequencing, Sprout tumble, Tuber production

## Abstract

**Background:**

*Pinellia ternata* is native to China and has been used as a traditional herb due to its antiemetic, antitussive, analgesic, and anxiolytic effects. When exposed to strong light intensity and high temperature during the reproductive growth process, *P. ternata* withers in a phenomenon known as “sprout tumble”, which largely limits tuber production. Shade was previously found to delay sprout tumble formation (STF); however, no information exists regarding this process at the molecular level. Hence, we determined the genes involved in tuber development and STF in *P. ternata.*

**Results:**

Compared to that with natural sun-light (control), shade significantly induced chlorophyll accumulation, increased chlorophyll fluorescence parameters including initial fluorescence, maximal fluorescence, and *qP*, and dramatically repressed chlorophyll a:b and *NPQ*. Catalase (CAT) activity was largely induced by shade, and tuber products were largely increased in this environment. Transcriptome profiles of *P. ternata* grown in natural sun-light and shaded environments were analyzed by a combination of next generation sequencing (NGS) and third generation single-molecule real-time (SMRT) sequencing. Corrections of SMRT long reads based on NGS short reads yielded 136,163 non-redundant transcripts, with an average N50 length of 2578 bp. In total, 6738 deferentially-expressed genes (DEGs) were obtained from the comparisons, specifically D5S vs D5CK, D20S vs D20CK, D20S vs D5S, and D20CK vs D5CK, of which, 6384 DEGs (94.8%) were generated from the D20S vs D20CK comparison. Gene annotation and functional analyses revealed that these genes were related to auxin signal transduction, polysaccharide and sugar metabolism, phenylpropanoid biosynthesis, and photosynthesis. Moreover, the expression of genes enriched in photosynthesis appeared to be significantly altered by shade. The expression patterns of 16 candidate genes were consistent with changes in their transcript abundance as identified by RNA-Seq, and these might contribute to STF and tuber production.

**Conclusion:**

The full-length transcripts identified in this study have provided a more accurate depiction of *P. ternata* gene transcription. Further, we identified potential genes involved in STF and tuber growth. Such data could serve as a genetic resource and a foundation for further research on this important traditional herb.

## Background

*Pinellia ternata* belongs to the Araceae family and is a perennial herb that is widely distributed in the eastern part of Asia. Further, both its wild and cultivated varieties are mainly found in China [1, 2]. Its tuber is the main medicinal part, and this has been frequently used in traditional Chinese medicine for thousands of years [3–5]. Many studies have revealed the complex components of *P. ternata*, such as alkaloids, organic acid, polysaccharose, proteins, and nucleosides [6–11]. Alkaloid has however been recognized as its main active ingredient and is believed to exert anticancer effects [12, 13]. Besides this property, the antiemetic, antitussive, analgesic, and anxiolytic effects of *P. ternata* are valuable for its use as a traditional Chinese medicine [9, 10, 14, 15]. As the lectin protein in the tuber of *P. ternata* displays toxic effects [16], the development of an eco-friendly biopesticide using the extracts of this plant and the breeding of disease-resistant varieties using agglutinin gene engineering are warranted [17–19]. Although the current demand for *P. ternata* is growing, sources of the plant are becoming increasingly scarce due to over-exploitation and the lack of large-scale cultivation.

*P. ternata* is sensitive to light intensity and temperature during growth. When exposed to strong light and high temperature, it rapidly withers, forming a “sprout tumble” [20]. The “sprout tumble” often appears in the summer and directly limits tuber production, which exacerbates the disparities between the supply and demand of *P. ternata* [21]. Hence, reducing sprout tumble formation (STF) is becoming a key procedure to increase tuber production. The rate and date of STF in *P. ternata* can be dramatically remitted under shading conditions during its reproductive growth period and this has been revealed at the physiological level [22–24]. To date, however, a report revealing this mechanism at the molecular level has not been presented, thereby warranting the need for a complete understanding of the effect of shade on STF.

With the rapid development of molecular biology and bioinformatics, gene discovery can be performed in non-model species, especially for non-sequenced medicinal plants [25–27]. For *P. ternata*, candidate genes might be involved in tuber development, and its high temperature-response has been verified via EST library sequencing [20]. Genes related to the ephedrine biosynthetic pathway were previously revealed by next-generation sequencing (NGS) [28]. Although NGS is currently used to generate large amounts of omics data [29, 30], identifying gene isoforms based on the short reads generated from NGS could be hindered by a high incidence of false positives [31]. Single-molecule real-time (SMRT) sequencing was recently demonstrated to offer a great advantage over NGS technologies in terms of read length [32] and has since been used to characterize the complexity of transcriptomes in *Arabidopsis pumila*, *Hordeum vulgare*, and *Camellia sinensis* [33–35]. As mentioned, the genes that regulate STF respond to shade, whereas those that regulate tuber production are largely unknown. In this study, we combined NGS and SMRT sequencing technologies to identify the full-length transcriptome response of *P. ternata* to shade, as well as candidate genes involved in STF and tuber development. This work serves as a foundation to functionally elucidate the genes involved in tuber development and STF in *P. ternata*.

## Results

### Shade inhibits the sprout tumble rate and promotes tuber production

**Fig. 1:**
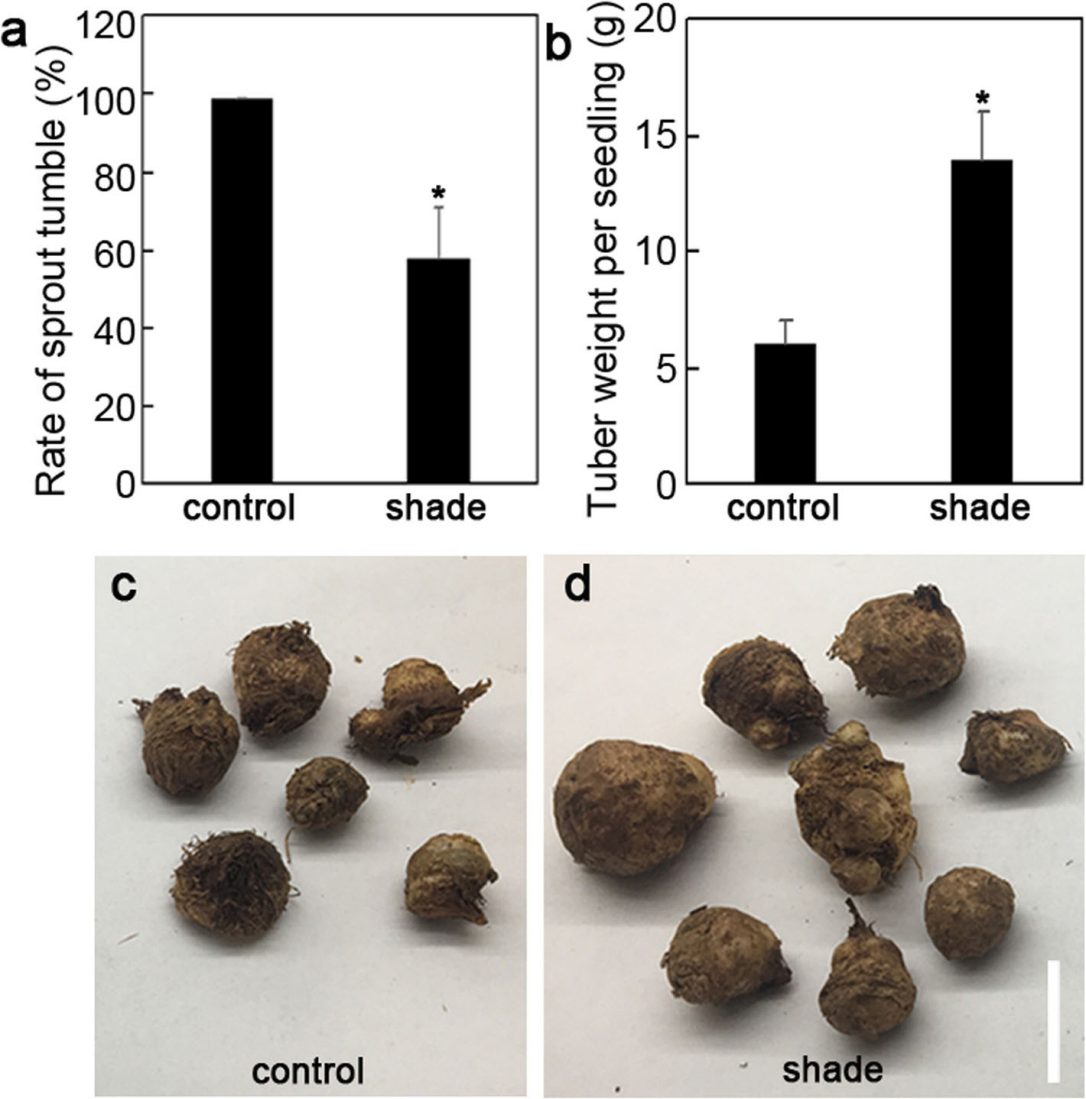
Effect of shade on the growth of *P. ternata*. Effect of shade on the rate of STF (**a**) and tuber weight (**b**) of *P. ternata* grown in a control and a shaded environment. Tuber weight of *P. ternata* grown in control and shade environment. Tubers harvested in control (**c**) and shaded environment (**d**) after all *P. ternata* had STF. Data are presented as mean ± SD (*n* > 30). * indicates significant difference (*P* < 0.05)

**Fig. 2:**
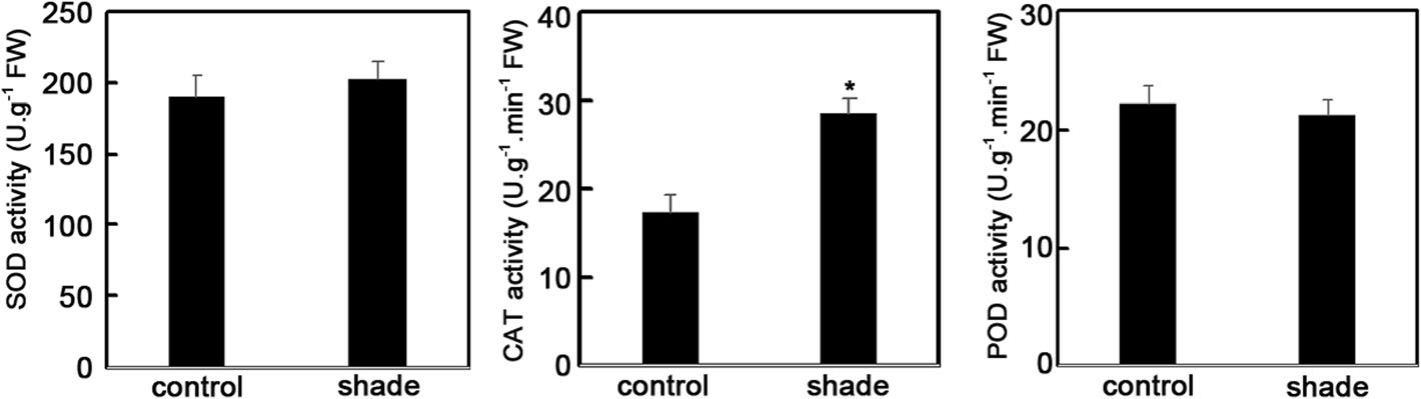
Effect of the 20-d-shade treatment on SOD, CAT, and POD enzymatic activity in *P. ternata*. Data are presented as mean ± SD (n > 30). * indicates significant difference (*P* < 0.05)

To investigate the effect of shade on *P. ternata*, we evaluated the rate of STF in *P. ternata* grown under control and shade conditions. Almost all *P. ternata* plants grown in control conditions showed STF, but this rate was sharply decreased in the shade (Fig. 1a). Meanwhile, the tubers generated from *P. ternata* grown in the shade weighed ~ 14 g per seedling, 2-fold greater than the control weight (Fig. 1 b and d). We next sought to detect the physiological traits of *P. ternata*, and as shown in Table 1, the contents of chlorophyll a and chlorophyll b increased while the chlorophyll a:b ratio decreased in the shade compared to those in the control. Values of initial fluorescence (*F*_*0*_), maximal fluorescence (*F*_*m*_), and *qP* were elevated in the shade environment, whereas *NPQ* was reduced (Table 1). We also evaluated the enzyme activity of superoxide dismutase (SOD), catalase (CAT), and polyamine oxidase (POD) under control and shade conditions. CAT activity was largely increased in *P. ternata* plants grown in the shade. However, statistical significance was not found for SOD and POD activities between control and shade conditions (Fig. 2).

**Table 1:** Photosynthetic parameter of *P. ternata* under control and shade environments

Photosynthetic parameter	Light condition	
	Control	Shade
Chlorophyll a content (mg. g^− 1^ DM)	3.07 ± 0.17	4.88 ± 0.32*
Chlorophyll b content (mg. g^−1^ DM)	1.05 ± 0.11	2.11 ± 0.18*
Chlorophyll (a + b) content (mg. g^−1^ DM)	4.12 ± 0.28	6.99 ± 0.49*
Chlorophylla:b	2.93 ± 0.15	2.32 ± 0.08*
Initial fluorescence (*F*_*0*_)	219.54 ± 13.61	247.33 ± 11.90*
Maximal fluorescence (*F*_*m*_)	1153.46 ± 162.29	1240.17 ± 6.39*
*qP*	0.91 ± 0.039	0.97 ± 0.012*
*NPQ*	0.027 ± 0.005	0.016 ± 0.008*

Data are presented as means ± sd from three replicates with 30 explants in each replicate. * indicates significant difference between control and shade (*P* < 0.05)

### Transcriptome sequencing

To identify and characterize the transcriptomes of *P. ternata,* between control and shade environments, NGS and SMRT sequencing technologies were employed for whole-transcriptome profiling. More than 617 million clean reads were generated by Illumina sequencing (Additional file 1: Table S1). In addition, SMRT generated 498,369 insert reads, of which 297,364 were full-length non-chimeric (flnc) reads and 127,681 were non-full-length reads (Table 2). The average flnc read length was 2306 bp (Additional file 2: Table S2).

**Table 2:** Statistics of SMRT sequencing data

Library	Number
Number of SMRT cells	2
Number of reads of insert	498,369
Number of 5′ reads	418,110
Number of 3′ reads	444,553
Number of ploy-A reads	430,421
Number of filtered short reads	21,211
Number of non-full-length reads	127,681
Number of full-length reads	349,477
Number of full-length non-chimeric reads	297,364
Average full-length non-chimeric read length	2306

**Fig. 3:**
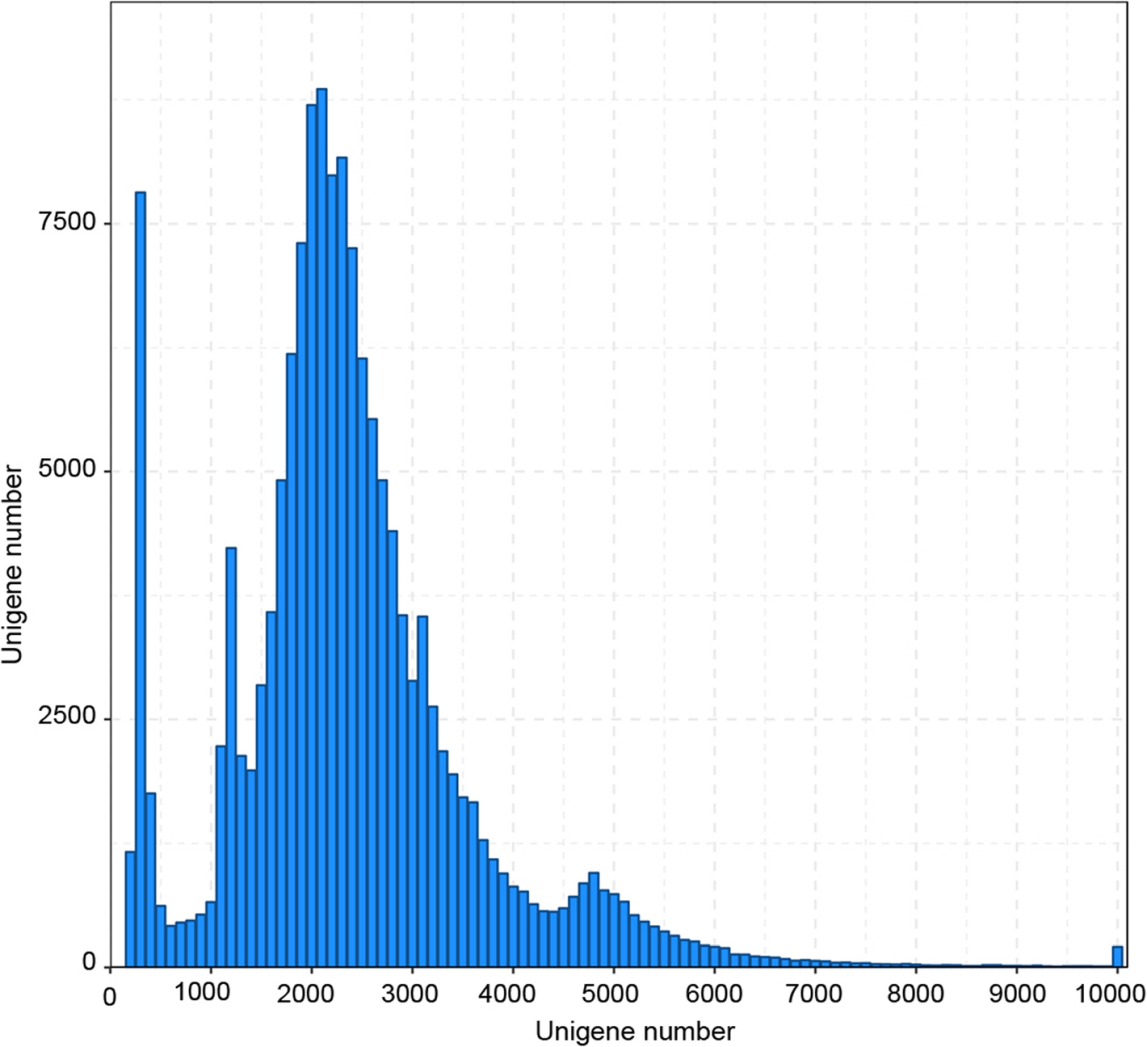
Length distribution of the *P. ternata* transcripts

To reduce the high error rates of the subreads, all SMRT reads were corrected using the ~ 617 million Illumina clean reads as input data (Additional file 1: Table S1). After error correction and the elimination of redundant transcripts using the CD-HIT program, 136,163 non-redundant transcripts were generated, each of which represented a unique full-length transcript with an average length of 2348 bp and N50 of 2578 bp (Additional file 2: Table S2). Approximately 83,075 unigenes (61%) were longer than 2 kb and only 13,074 unigenes (9.6%) were shorter than 500 bp (Fig. 3). Altogether, the SMRT sequencing technology provided a high-quality transcript profile with many full-length genes in *P. ternata*.

### Functional annotation and categorization of genes

To acquire a comprehensive annotation profile, NCBI non-redundant protein (NR), Kyoto Encyclopedia of Genes and Genomes (KEGG), Protein family (Pfam), Swiss-Prot, EuKaryotic Ortholog Groups (KOG), Gene Ontology (GO) by BLASTX, and NCBI nucleotide sequences (NT) by BLASTN were used to align the 13,613 transcripts. A total of 115,708 (84.98%) genes were successfully annotated using these databases. A Venn diagram showed that 43,106 genes were simultaneously annotated by NR, GO, Pfam, KOG, and NT (Additional file 3: Fig. S1A). Based on the alignment of sequences from different species in the NR database, 79,607 (68.8%) sequences had significant hits for *Anthurium amnicola*, followed by *Elaeis guineensis* (5207, 4.5%), *Phoenix dactylifera* (3703, 3.2%), *Nelumbo nucifera* (2314, 2.0%), and *Ananas comosus* (2083, 1.8%). Only 19.7% of the annotated sequences were identified based on sequences in other plant species (Additional file 3: Fig. S1B).

Various TF families including the WRKY, NAC, and MYB families were confirmed to be involved in the regulation of plant growth and their response to the environment. Transcriptome analysis of *P. ternata* revealed that 7783 genes (5.7%) encode putative TFs that can be classified into the 28 major TF families, as well as some other families. Members of the C3H, WRKY, and bHLH TF families occupied the top three slots, and all exceeded 400. The number of GRAS, AP2/ERF, bZIP, C2H2, NAC, MYB-related, HSF, and B3 families exceeded 200 (Additional file 4: Fig. S2). The identification of this large set of TFs provides a rich resource for the further analysis of specific TFs in various life processes of *P. ternata.*

To functionally classify *P. ternata* genes, they were mapped to various terms of the GO database (http://www.geneontology.org/). A total of 73,250 genes were mainly classified into three major categories (biological process, cellular component, and molecular function). Based on molecular function classification, major categories were “binding” and “catalytic activity”. The major subgroups for cellular components were “cell”, “cell part”, “organelle”, “membrane”, and “membrane part”. For the biological process category, genes involved in the “metabolic process”, “cellular process”, and “single-organism process” were highly represented (Additional file 5: Fig. S3).

To understand the functions in a specific metabolic pathway of *P. ternata*, we assigned the assembled genes to KEGG biological pathways. Consequently, functions could be assigned to five main categories as follows: “Cellular Processes”, “Environmental Information Processing”, “Genetic Information Processing”, “Metabolism”, and “Organismal Systems”, with 33 sub-categories. Among these categories, “signal transduction” had 6757 genes, “carbohydrate metabolism” had 5925 genes, and “endocrine system” had 3462 genes, which provides valuable information for further gene function analysis (Additional file 6: Fig. S4).

### Analysis of differentially-expressed genes (DEGs)

To evaluate gene expression levels in response to shade, Illumina clean reads of control and shade samples assembled with the SMRT full-length transcriptome were mapped. Thereafter, read-counts for each gene were obtained from the mapping results, and then converted into an expected number of fragments per kilobase of transcript sequence per million base pairs (FPKM).

**Fig. 4:**
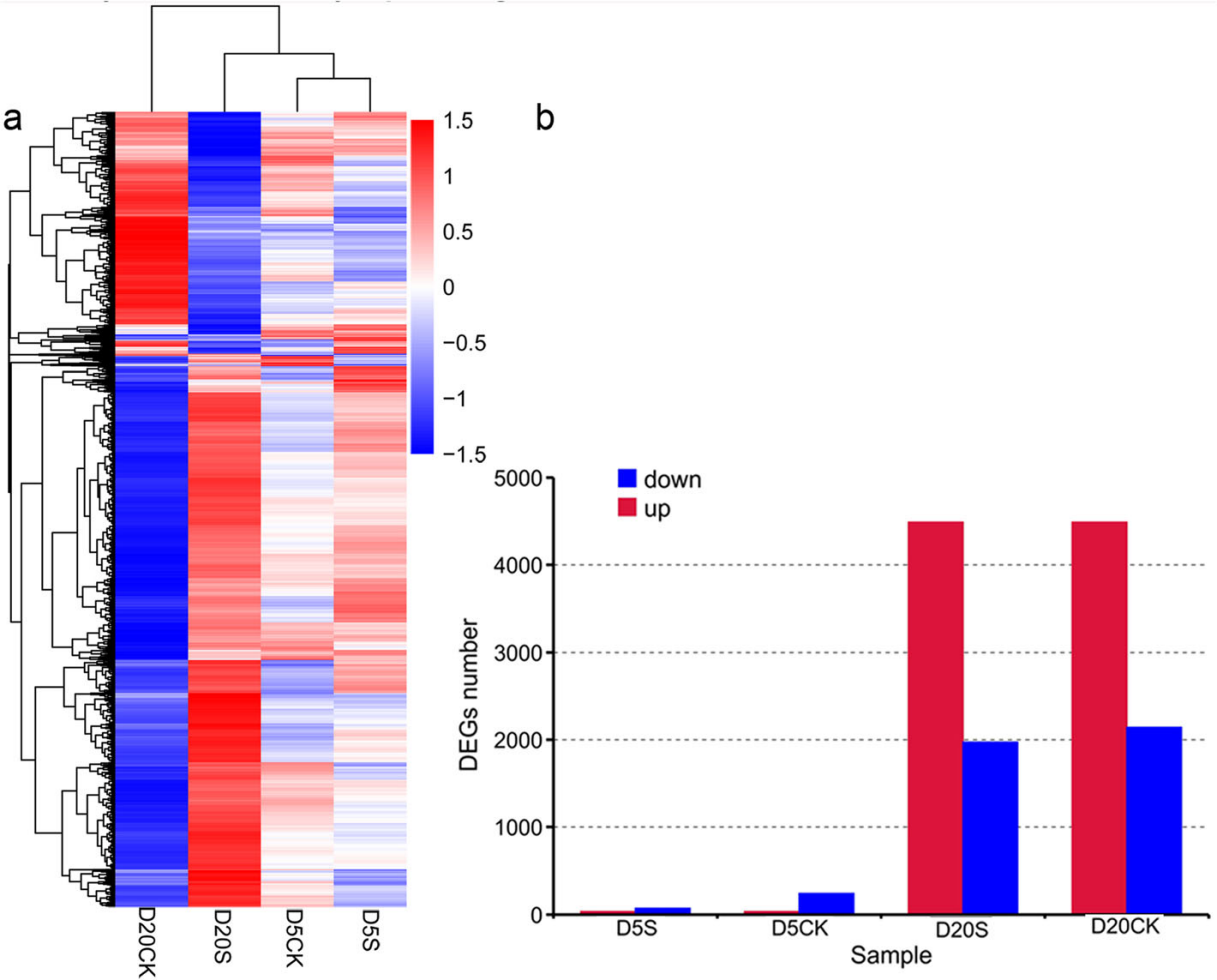
Clustering analysis of the DEGs. **a** Hierarchical clustering graph of the 6738 DEGs based on average log_10_(FPKM+ 1) values of all genes in each cluster. **b** statistics of upregulated- and downregulated-expression of DEGs in each cluster

In total, 6738 DEGs that were up or downregulated between samples (*P* < 0.05) were collected. Clustering patterns of DEGs under different experimental treatments were determined by the cluster analysis of all DEGs with the Euclidean distance method and complete linkage (Fig. 4). This clustering pattern suggested that many genes were activated or inhibited in the D20S vs D20CK comparison, and the number of DEGs were much greater than those with the D5S vs D5CK, D20S vs D5S, and D20CK vs D5CK comparisons (Additional file 7: Fig. S5).

**Fig. 5:**
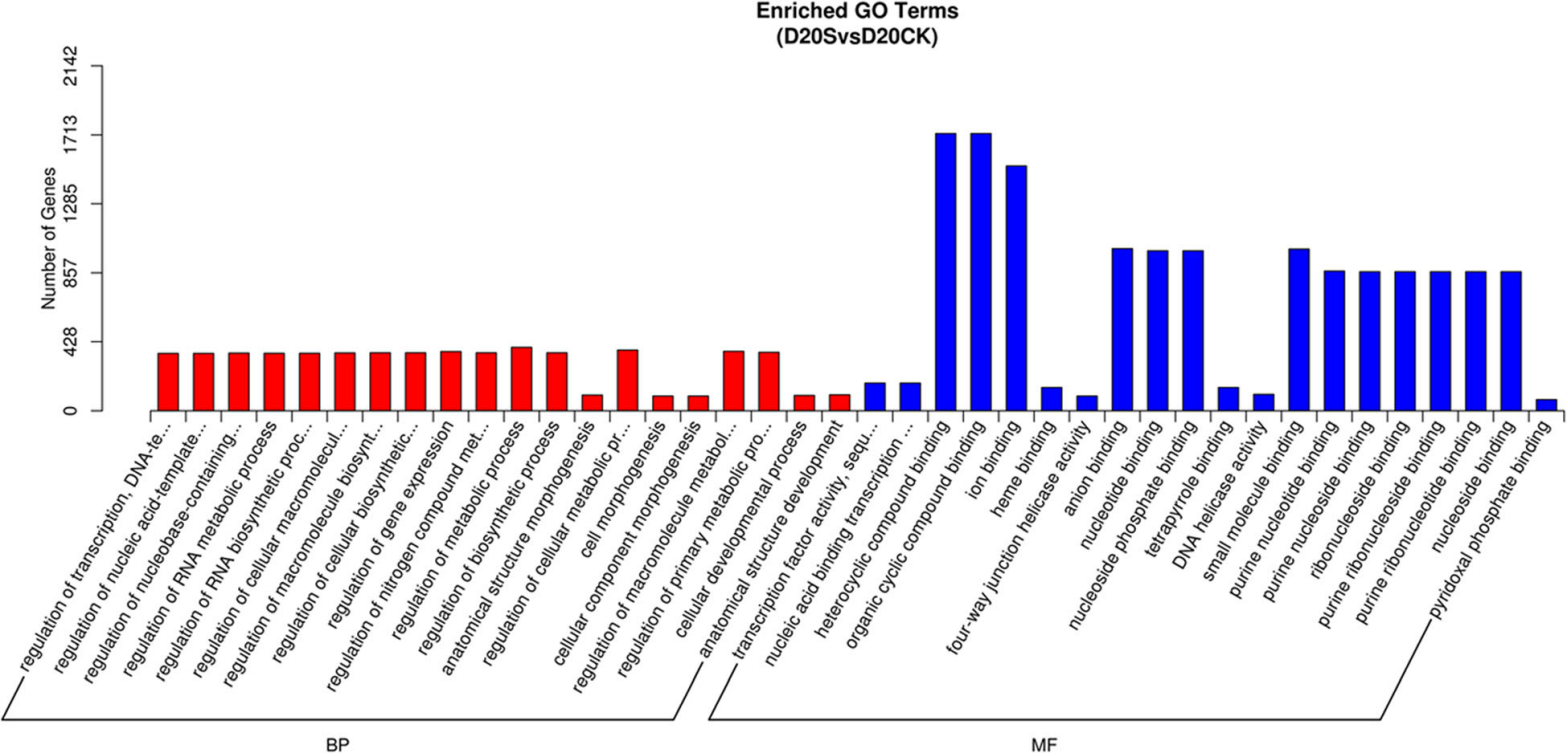
Enriched GO Terms of DEGs generated from the D20S vs D20CK group. BP and MF are abbreviations of biological process and molecular function, respectively

**Fig. 6:**
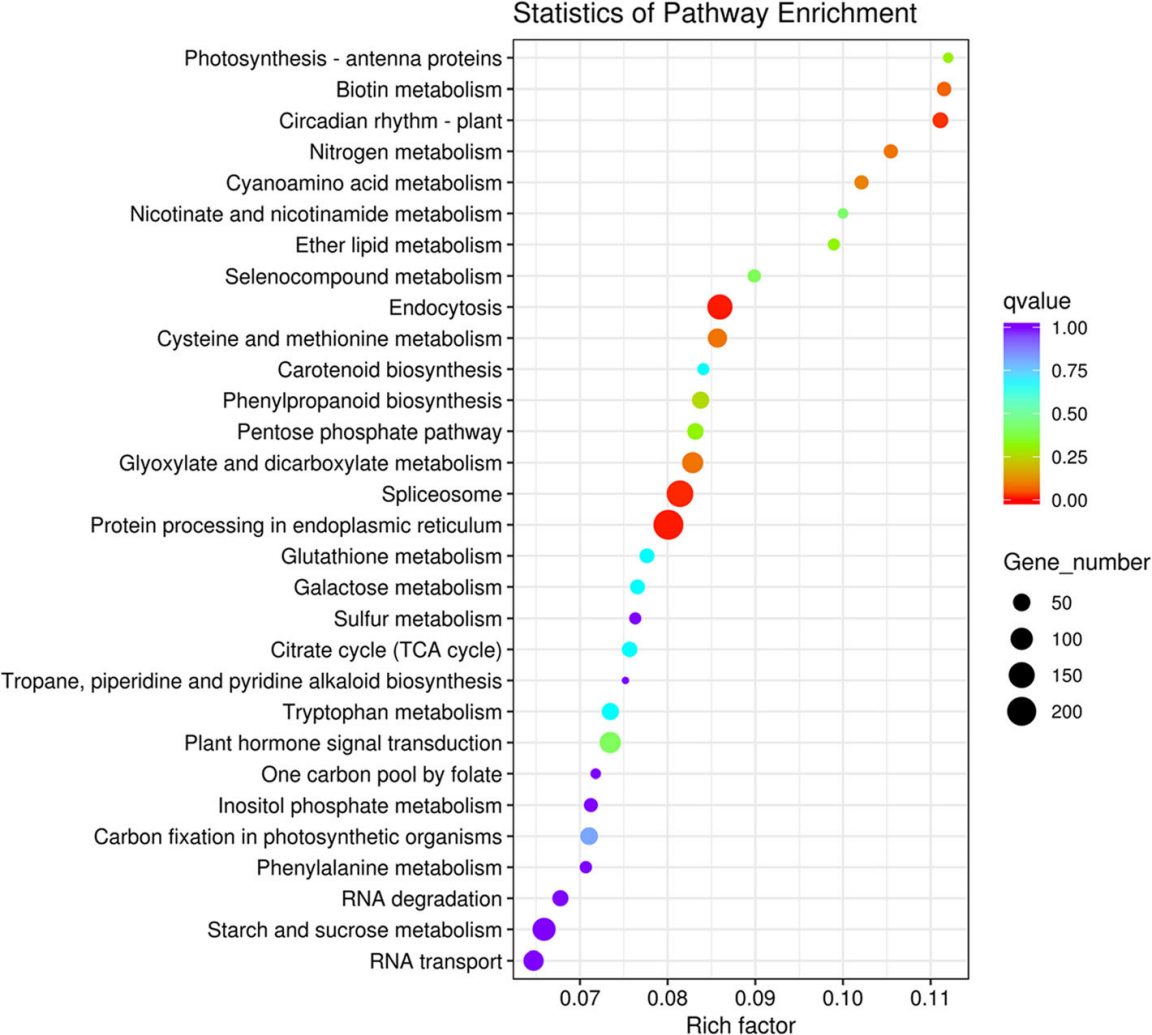
KEGG enrichment analyses with the DEGs generated from the D20S vs D20CK group. The rich factor is the ratio of differentially expressed genes versus all annotated genes in corresponding pathways

To gain a better understanding of DEG functions, we selectively analyzed the 6384 DEGs from the D20S vs D20CK comparison. Based on GO functional enrichment analysis, these DEGs could be classified into two categories, namely biological processes and molecular functions, with 30 functional groups. For the molecular function ontology, the dominant terms were “heterocyclic compound binding”, “organic cyclic compound binding”, and “ion binding”. For biological processes, 15 functional groups had almost equivalent gene numbers without dominant terms (Fig. 5). KEGG pathway enrichment analysis was also carried out and DEGs could be assigned to 112 KEGG pathways. Here, we focused on the top 30 pathways and screened four that are closely related to STF and tuber growth, namely “photosynthesis-antenna proteins”, “phenylpropanoid biosynthesis”, “plant hormone signal transduction”, and “starch and sucrose metabolism” (Fig. 6).

### qRT-PCR validation of candidate genes involved in STF and tuber growth

**Fig. 7:**
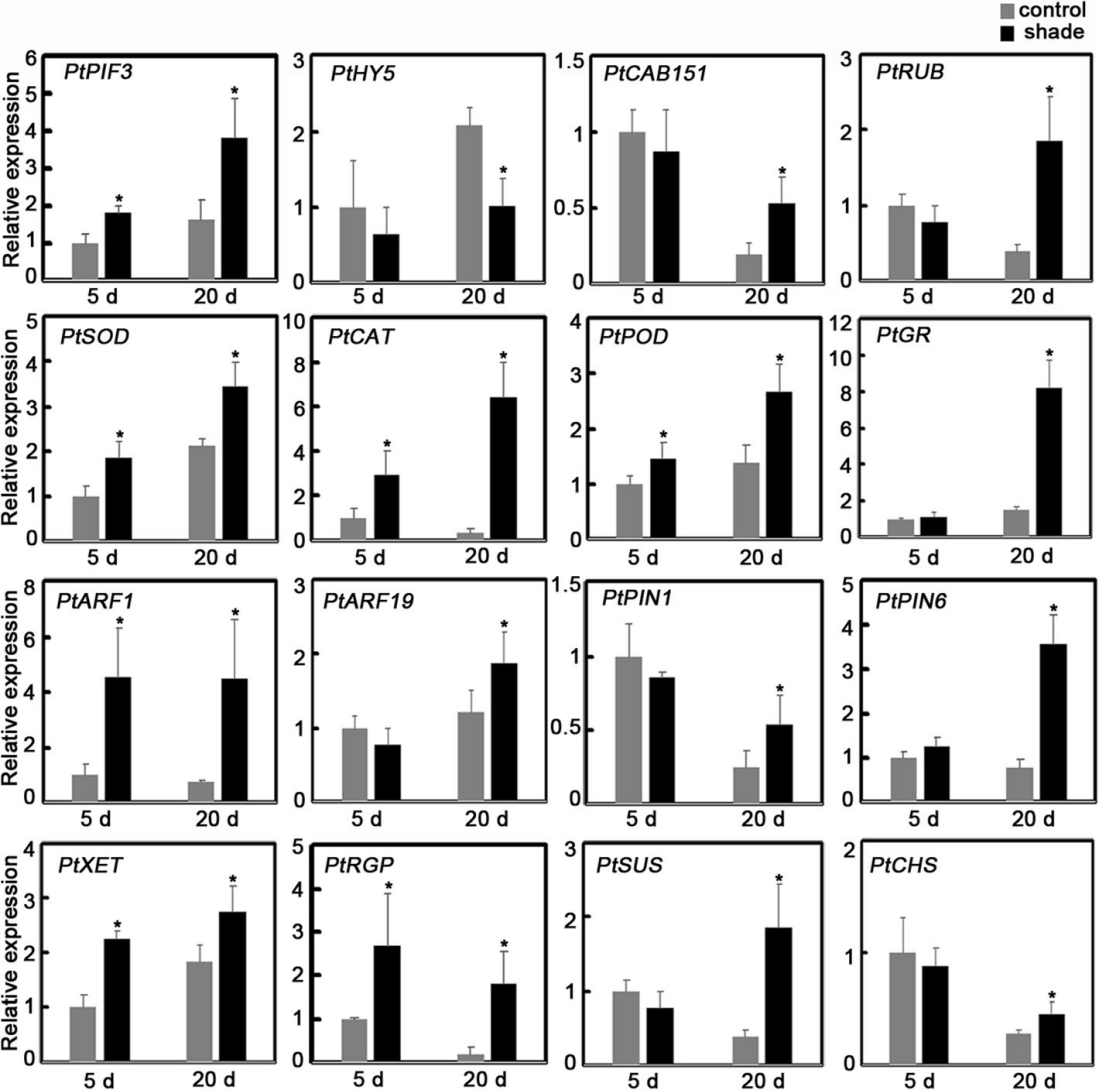
The 16 genes with differential expression when *P. ternata* is grown for 5 and 20 d in control and shaded environment, respectively. All the data represent the values relative to those of control at 5d. Data are presented as mean ± SD. * means differed significantly (*P* < 0.05). PIF3: phytochrome-interacting factor 3; HY5: elongated hypocotyls 5; CAB151: chlorophyll a/b-binding proteins 151; RUB: rubisco; SOD: superoxide dismutase; CAT: catalase; POD: polyamine oxidase; GR: glutathione reductase; ARF1/19: auxin response factor 1/19; PIN1/6: PIN FORMED1/6; XET: β-xyloglucan endotransglycosylase; RGP: reversible glycosylated polypeptides; SUS: sucrose synthase; and CHS: chalcone synthase

To validate the transcriptome data, 16 candidate DEGs that could be associated with STF and tuber growth were selected for qRT-PCR analysis. As shown in Fig. 7, DEGs involved in antioxidant protection (*SOD*, *CAT*, *POD*, and *GR*), auxin signal transduction (*ARF1*, *ARF19*, *PIN1*, and *PIN6*), polysaccharide and sugar metabolism (*XET*, *RGP*, and *SUS*), and phenylpropanoid biosynthesis (*CHS*) were upregulated. DEGs related to photosynthesis (*PIF3*, *CAB151*, and *RUB*) were also upregulated but *HY5* was downregulated in the 20-d-shade environment. Moreover, fold-changes in their expression, which were calculated by sequencing did not match the expression data detected by qRT-PCR. However, the expression trends were in agreement for these 16 genes. We also analyzed expression of these genes after growing *P. ternata* in the shade environment for 5 d. Compared to control levels, the expression of most genes showed no significant changes, although several genes (*PtPIF3*, *PtSOD*, *PtCAT*, *PtPOD*, *PtARF1*, *PtXET*, and *PtRGP*) were upregulated (Fig. 7). The expression profiles thus confirmed the reliability of the transcriptomic data and provided useful information to understand STF and tuber growth in *P. ternata*.

## Discussion

Plant growth and development often correlate with environmental factors. Two important factors, light and temperature, always regulate similar developmental processes in the lifecycle of plants [36, 37]. High temperature along with strong light often causes STF in *P. ternata* [20]. Here, we revealed that growth in a shaded environment promotes tuber production by inhibiting the rate of STF, which aligns with the findings of a previous report [22].

Recently, some researchers assessed *P. ternata* and explored some of its transcript profiles for high-temperature stress [20], in vitro tuber induction [37], and the ephedrine biosynthetic pathway [28]. However, the genetic background remains largely unknown, thereby prohibiting further research and improvements in *P. ternata* cultivation. Currently, the use of a hybrid sequencing approach where NGS and SMRT are combined is gradually increasing, which could provide high-quality and complete assemblies, especially with non-sequenced species [38–40]. In this study, a more complete transcriptome of *P. ternata* was generated by using NGS and SMRT technologies. Corrections of the SMRT reads using the NGS reads generated high-quality, full-length reads, largely reducing the mis-assemblies of genes with high sequence identity. Such high-quality data have aided in the understanding of STF and tuber growth and have allowed for future studies on *P. ternata*.

By comparing DEGs generated in different groups, we found that DEGs in the D20S vs D20CK comparison accounted for 94.12% (Additional file 7: Fig. S5). This result suggests that a long duration of shade treatment might be the key factor promoting tuber development through the altered expression of many genes. The 112 KEGG pathways involved in DEGs revealed that STF and tuber growth in *P. ternata* are intricate life phenomena (Fig. 6). In the shaded condition, photosynthetic traits of *P. ternata* including chlorophyll content and chlorophyll fluorescence parameters were significantly increased (Table 1), demonstrating that this condition improves photosynthesis, which aligns with previous data obtained in our lab [41]. Similarly, the transcript of *PtPIF3*,which encodes a suppressor of photosynthesis, was found to increase while that of the positive regulator *PtHY5* decreased after 20 d of shade treatment (Fig. 7) [42]. This indicates that the light signaling pathway is greatly altered in the shade environment. Furthermore, the transcript abundances of *PtCAB151* and *PtRUB* markedly increased in the 20-d shade environment (Fig. 7) [43, 44]. Therefore, by combining physiological and genetic data, photosynthetic efficiency was found to increase to capture the limited light resource [45, 46]; this result aligns with that of a previous study where soybean photosynthesis was found to be enhanced in a shaded environment [47]. Only *PtPIF3* was upregulated in *P. ternata* after 5 d of shade treatment. This demonstrated that the photosystem of *P. ternata* was affected at day 5 and was amplified after a 20-d shade treatment.

Higher plants use light energy for photosynthesis. However, this always leads to photoinhibition when the light energy absorbed by pigments exceeds the capacity of the photosynthetic apparatus [48]. This can then lead to ROS production, which damages the photosynthetic system [49, 50]. Based on oxidative stress, related-enzymes, mainly SOD, CAT, POD, and glutathione reductase (GR), can be induced to eliminate ROS [50–54]. Previous data demonstrated that *P. ternata* easily underwent photoinhibition when it was exposed to strong light in the summer [55]. Here, transcripts of *PtSOD*, *PtCAT*, and *PtPOD* were increased after a 5-d shade treatment, and the expression of *PtSOD*, *PtCAT*, *PtPOD*, and *PtGR* were upregulated after a 20-d shade treatment (Fig. 7). The activity of *Pt*CAT dramatically increased in the shaded environment, whereas the activities of *Pt*SOD and *Pt*POD showed no significant changes (Fig. 2). These findings imply that shading treatment protects *P. ternata* from ROS damage by regulating CAT activity, which delays STF. It was speculated that the activities of *Pt*SOD and *Pt*POD were further regulated at the translation level, thereby reducing the differences in genes expression, as required for precise regulation. Notably, CHS is a key enzyme in flavonoid synthesis and it is involved in the phenylalanine synthesis pathway and the regulation of plant resistance to adversity [56]. Our study showed that the expression level of *PtCHS* was strongly induced in the 20-d shade environment (Fig. 7), aligning with a previous report showing that light suppresses *PtCHS* accumulation, thereby inhibiting sweet potato growth [57]. The implication is that high *PtCHS* expression might promote tuber growth in *P. ternata*; however, whether it participates in the regulation of STF requires further verification.

**Fig. 8:**
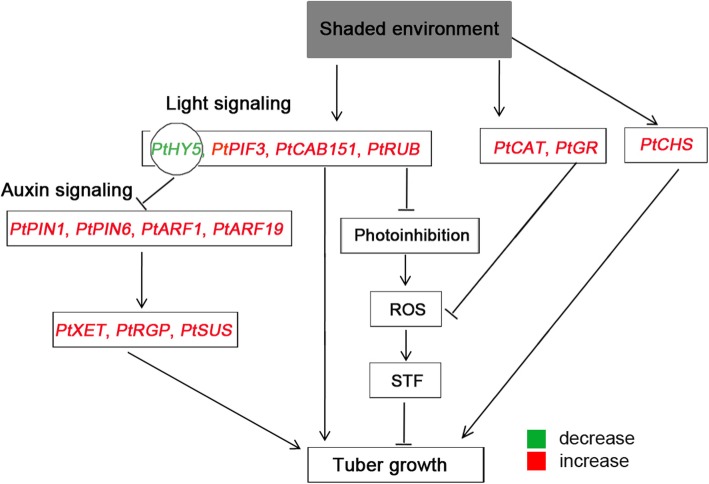
Putative gene interaction model for tuber growth of *P. ternata* in a shaded environment. A shaded environment suppresses light signaling and increases the photosynthetic efficiency by repressing *PtHY5* expression and increasing the transcripts of *PtPIF3*, *PtCAB151*, and *PtRUB*, which not only promote tuber growth directly, but also reduces ROS content via remitting photoinhibition, thereby delaying STF and facilitating tuber growth indirectly. The reduction in *PtHY5* expression causes auxin signaling by up-regulating the expression of *PtPIN1*, *PtPIN6*, *PtARF1*, and *PtARF19* which further increases the expression of *PtXET*, *PtRGP*, and *PtSUS*, accelerating tuber growth. Additionally, the induction of *PtCAT* and *PtGR* in a shaded environment promotes tuber growth by indirectly eliminating ROS accumulation while the up-regulation of *PtCHS* expedites tuber growth

Through exposure to a shaded environment, *P. ternata* plants grow faster, with high seedling heights and enlarged leaf and tuber size [22]. Plant growth requires cell proliferation and enlargement, which are closely-related to cell wall loosening and rebuilding. In addition, changes in light signaling perception were found to exhibit cross-talk with hormone-mediated plant growth regulation pathways [38]. *Pt*HY5 was proven to function as a negative regulator that links light and hormone signals. Hence, the decreased accumulation of *PtHY5* in a shaded environment could result in an intense auxin signal, which is consistent with published data that shading increases IAA content in *P. ternata* [22, 36]. Currently, many genes involved in IAA regulation in a shaded environment were identified in a model plant. A previous study reported that the auxin transporter PIN3 and the TF families Aux/IAA and ARF are induced by shade [58]. The expression of *PtARF1*/*19* and *PtPIN1*/*6* were significantly increased in a shaded environment (Fig. 7), which also confirmed that the auxin signal was invigorated by shading. β-xyloglucan endotransglycosylase (XET) usually mediates cell wall loosening by modifying the xyloglucan chains. In contrast, reversible glycosylated polypeptides (RGPs) function in cell wall synthesis by participating in polysaccharide biosynthesis [59, 60]. Thus, the expression of *PtXET* and *PtRGP* were positively correlated with tissue expansion. The upregulation of *PtXET* and *PtRGP* genes suggested that cell wall biosynthesis is stimulated by shade (Fig. 7). Besides cell wall reconstitution, cell enlargement also requires many protoplasmic substances. Starch is the main storage-carbohydrate in plants, especially for harvesting organs containing tubers. Sucrose synthase (SUS) is one of the key enzymes that promote the entry of sucrose into various metabolic pathways, and mainly the positive regulation of starch synthesis and plant stress resistance [61, 62]. Hence, upregulation of the *PtSUS* gene causes starch accumulation in tubers. Overall, we can conclude that the shade environment increases photosynthetic efficiency and reduces ROS accumulation, leading to a decrease in the STF rate. Combinations of polysaccharide metabolic mechanisms including those related to cell wall and starch were induced by shade. Together, they account for the promotion of tuber growth in the shade (Fig. 8).

## Conclusions

Herein, we generated a comprehensive transcriptome based on the response of *P. ternata* plants to a shaded environment using NGS and MGRT sequencing technologies. A total of 136,163 non-redundant transcripts were generated and 6738 DEGs involved in many KEGG pathways were identified. Sixteen DEGs were selected for expression analysis. Shade was found to improve photosynthetic efficiency and protective enzyme activity, ultimately avoiding photoinhibition-induced ROS damage and causing a delay in STF. Auxin signals and enzymatic genes involved in cell wall reconstruction were upregulated, thereby inducing cell enlargement. These findings add to our knowledge of STF and tuber growth at the molecular level. Moreover, our study provides the first full-length transcriptome resource for *P. ternata* and lays a foundation for further research on this important traditional herb.

## Methods

### Plant materials

The original *P. ternata*, identified by Prof. Jianping Xue (Huaibei Normal University), were obtained from the wild without any specifically permissive requirement and the plant samples were deposited in the specimen room of Huaibei Normal University. It is cultivated in the Experimental Farm of Huaibei Normal University in Huaibei City, Anhui Province, P.R. China (33°16′ N, 116°23′ E, altitude, 340 m). No specific permissions were required for samples collection. We stated that the field studies were in accordance with local legislation and no specific licences were required. The tubers of *P. ternata* were planted in the Experimental Farm of Huaibei Normal University in May 2016. When plants grew, approximately 3 weeks after sprouting, half were treated with ~ 90% shade. The rates of sprout tumble were calculated by analyzing the growth status of *P. ternata* in three random shade- and light-zones after a 30-d shade-treatment. Tuber production per seedling was calculated by measuring the weight of tubers of 30 seedlings in the shade and light zones after *P. ternata* had exhibited STF. Three random shade and light zones were selected for statistical analysis.

### Photosynthetic parameters and detection of antioxidant protective enzyme activity

Leaves of *P. ternata* grown in shade and light zones for 20 d were used to detect photosynthetic parameters including the content of chlorophyll a and chlorophyll b, *F*_*0*_, *F*_*m*_, *qP*, and NPQ using a previously described method [23]. SOD, CAT, and POD activities in these materials were measured with reference to protocols published previously by our lab [63].

### RNA isolation, Illumina cDNA library preparation, and NGS

*P. ternata* seedlings were grown in natural light and in a shaded environment and were sampled at 5 and 20 d (i.e. D5CK, D5S, D20CK, and D20S, respectively). The experiments contained three biological replications. Total RNA of the 12 samples was extracted for transcriptome sequencing using a protocol described previously [64]. Sequencing libraries were generated using a NEBNext® Ultra™ RNA Library Prep Kit for Illumina® (NEB) following the manual’s instruction. NGS was performed at the Novogene Bioinformatics Institute (Novogene, Beijing, China).

### PacBio Iso-Seq library preparation, SMRT sequencing and data processing

Twelve RNA samples were mixed together with equal quantity, and then the mixed RNA was used to prepare SMRT libraries according to the protocol described by Yang et al. [33]. Subsequently, the libraries were sequenced with a Pacific Biosciences RS sequencing instrument. Sequence data were processed using SMRTlink 5.0 software. Circular consensus sequence (CCS) was generated from subread BAM files, and unigenes were obtained using the method reported by Song et al. [64].

### Functional annotation and classification

Gene function was annotated based on the following databases: NR (NCBI non-redundant protein sequences), NT (NCBI non-redundant nucleotide sequences), Pfam (http://pfam.xfam.org/), KOG/COG (http://www.ncbi.nlm.nih.gov/COG/), Swiss-Prot (http://www.expasy.ch/sprot), KO (KEGG Ortholog database), and GO (Gene Ontology). The Blast2GO program (http://www.blast2go.com) was used to annotate GO functional classifications. KEGG classification maps were generated based on the retrieved Kyoto Encyclopedia of Genes and Genomes Orthology (KO) information (http://www.genome.jp/kegg). Plant transcription factors in *P. ternata* were identified using the plant transcription factor database, PlantTFDB 4.0.

### Identification and functional analysis of DEGs

Differential expression analysis was performed with the DESeq R package (1.10.1) to identify DEGs between samples from the shade and control environments. The expression of DEGs with adjusted *P*-values < 0.05 based on DESeq were screened as deferentially-expressed, and an absolute log_2_ (Group1 / Group2) value ≥1 was used as the threshold to identify significant DEGs between different groups. GO enrichment analysis of DEGs was performed using the GOseq R packages. KOBAS software was used to test the statistical enrichment of DEGs in the KEGG pathways.

### Validation of DEGs using qRT-PCR

qRT-PCR assays were performed to validate the 16 candidate DEGs related to STF and tuber growth. cDNA synthesis and transcript abundance analysis were performed with a protocol described previously [65]. Primer sequences are listed in Additional file 8: Table S3. The *P. ternata* 18S gene was used as an internal control. Three biological replicates and three technical repeats were used for each gene and sample. The relative mRNA expression level was calculated based on the 2^−ΔΔCT^ method.

### Statistical analysis

All assays were conducted in triplicate, the SPSS statistical software 20.0 (SPSS Inc., Chicago, USA) was employed for statistical analyses. The Student’s test was applied to test the significant differences between two samples at the level of *P* < 0.05.

## Supplementary information

**Additional: file 1 Table S1.** Overview of sequence data quality obtained from Illumina sequencing.

**Additional file 2: Table S2.** Summary of final *P. ternata* nonredundant transcripts generated from SMRT.

**Additional file 3: Fig. S1** Functional annotation and categorization of *P. ternata* transcripts. a Venn diagram of NR, NT, GO, KOG and Pfam results for the *P. ternata* transcripts. b Homologous species of *P. ternata* transcripts.

**Additional file 4: Fig. S2** Number and family of top 29 TFs predicted by SMRT.

**Additional file 5: Fig. S3** Distribution of GO terms for all annotated transcripts in biological process, cellular component and molecular function.

**Additional file 6: Fig. S4.** KEGG pathways enriched of transcripts.

**Additional file 7: Fig. S5.** Volcano map-analysis of differential expression genes.

**Additional file 8: Table S3.** Primers used for real-time quantitative PCR.

## Data Availability

All relevant data are available within the manuscript. The raw bam files of the Sequence Read Archives (SRA) were deposited at the National Center for Biotechnology Information under the accession number SRP215828. The PacBio SMRT reads used for this study were deposited at the National Center for Biotechnology Information Sequence Read Archive under the accession number PRJNA515824. The data can be access with the identifiers search in the web-link (https://www.ncbi.nlm.nih.gov/Traces/study).

## Abbreviations

ARF1/19: Auxin response factor 1/19
CAB151: Chlorophyll a/b-binding proteins 151
CAT: Catalase
CHS: Chalcone synthase
DEGs: Differentially-expressed genes
flnc: full-Length non-chimeric
FPKM: Fragments per kilobase per million reads
GR: Glutathione reductase
HY5: Elongated hypocotyls 5
NGS: Next generation sequencing
PIF3: Phytochrome-interacting factor 3
PIN1/6: PIN FORMED1/6
POD: Polyamine oxidase
RGP: Reversible glycosylated polypeptides
ROS: Reactive oxygen species
RUB: Rubisco
SMRT: Single-molecule real-time
SOD: Superoxide
STF: Sprout tumble formation
SUS: Sucrose synthase
XET: β-xyloglucan endotransglycosylase
