# Supplementary material for: Full-length transcriptome analysis of shade-induced promotion of tuber production in Pinellia ternata
Source: BMC Plant Biol. 2019 Dec 18;19:565. doi: 10.1186/s12870-019-2197-9 (PMC6921527; doi:10.1186/s12870-019-2197-9)
Supplement: Supplementary file 1 — Additional: file 1 Table S1. Overview of sequence data quality obtained from Illumina sequencing. [file 12870_2019_2197_MOESM1_ESM.docx]

**Additional file 1: Table S1.** Overview of sequence data quality obtained from Illumina sequencing.

| **Samples** | **Raw Reads** | **Clean Reads** | **Clean Bases** | **Error (%)** | **Q20 (%)** | **Q30 (%)** | **GC Content (%)** |
| --- | --- | --- | --- | --- | --- | --- | --- |
| D5CK_1 | 46981942 | 45959612 | 6.89G | 0.03 | 97.12 | 92.41 | 56.60 |
| D5CK_2 | 54584056 | 53161928 | 7.97G | 0.03 | 97.18 | 92.48 | 56.30 |
| D5CK_3 | 50640698 | 49722616 | 7.46G | 0.03 | 97.45 | 93.14 | 54.37 |
| D5S_1 | 38377508 | 36795066 | 5.52G | 0.03 | 97.20 | 92.68 | 56.05 |
| D5S_2 | 54565662 | 51363508 | 7.7G | 0.03 | 97.33 | 93.11 | 56.47 |
| D5S_3 | 56862522 | 54906618 | 8.24G | 0.03 | 96.39 | 90.93 | 56.11 |
| D20CK_1 | 62465336 | 61440442 | 9.22G | 0.03 | 97.17 | 92.51 | 55.97 |
| D20CK_2 | 60074008 | 58320880 | 8.75G | 0.03 | 97.63 | 93.69 | 54.95 |
| D20CK_3 | 59358006 | 58426536 | 8.76G | 0.03 | 97.41 | 93.06 | 55.04 |
| D20S_1 | 50612284 | 48862528 | 7.33G | 0.03 | 97.06 | 92.53 | 55.51 |
| D20S_2 | 52502382 | 51563336 | 7.73G | 0.03 | 96.96 | 92.02 | 56.16 |
| D20S_3 | 48103528 | 46483724 | 6.97G | 0.03 | 95.45 | 89.38 | 55.09 |
| Total | 635127932 | 617006794 | 92.54G |  |  |  |  |
