# Supplementary material for: Full-length transcriptome analysis of shade-induced promotion of tuber production in Pinellia ternata
Source: BMC Plant Biol. 2019 Dec 18;19:565. doi: 10.1186/s12870-019-2197-9 (PMC6921527; doi:10.1186/s12870-019-2197-9)
Supplement: Supplementary file 2 — Additional file 2: Table S2. Summary of final P. ternata nonredundant transcripts generated from SMRT. [file 12870_2019_2197_MOESM2_ESM.doc]

**Additional file 2: Table S2.** Summary of final *P. ternate* non-redundant transcripts generated from SMRT.

| **Sample** | **Non-redundant transcripts** | **Total bases (bp)** | **Mean length (bp)** | **N50 (bp)** | **Average flnc read length** |
| --- | --- | --- | --- | --- | --- |
| Total | 136163 | 162510654 | 2348 | 2578 | 2306 |
