# Supplementary figures and images for: Full-length transcriptome analysis of shade-induced promotion of tuber production in Pinellia ternata
Source: BMC Plant Biol. 2019 Dec 18;19:565. doi: 10.1186/s12870-019-2197-9 (PMC6921527; doi:10.1186/s12870-019-2197-9)

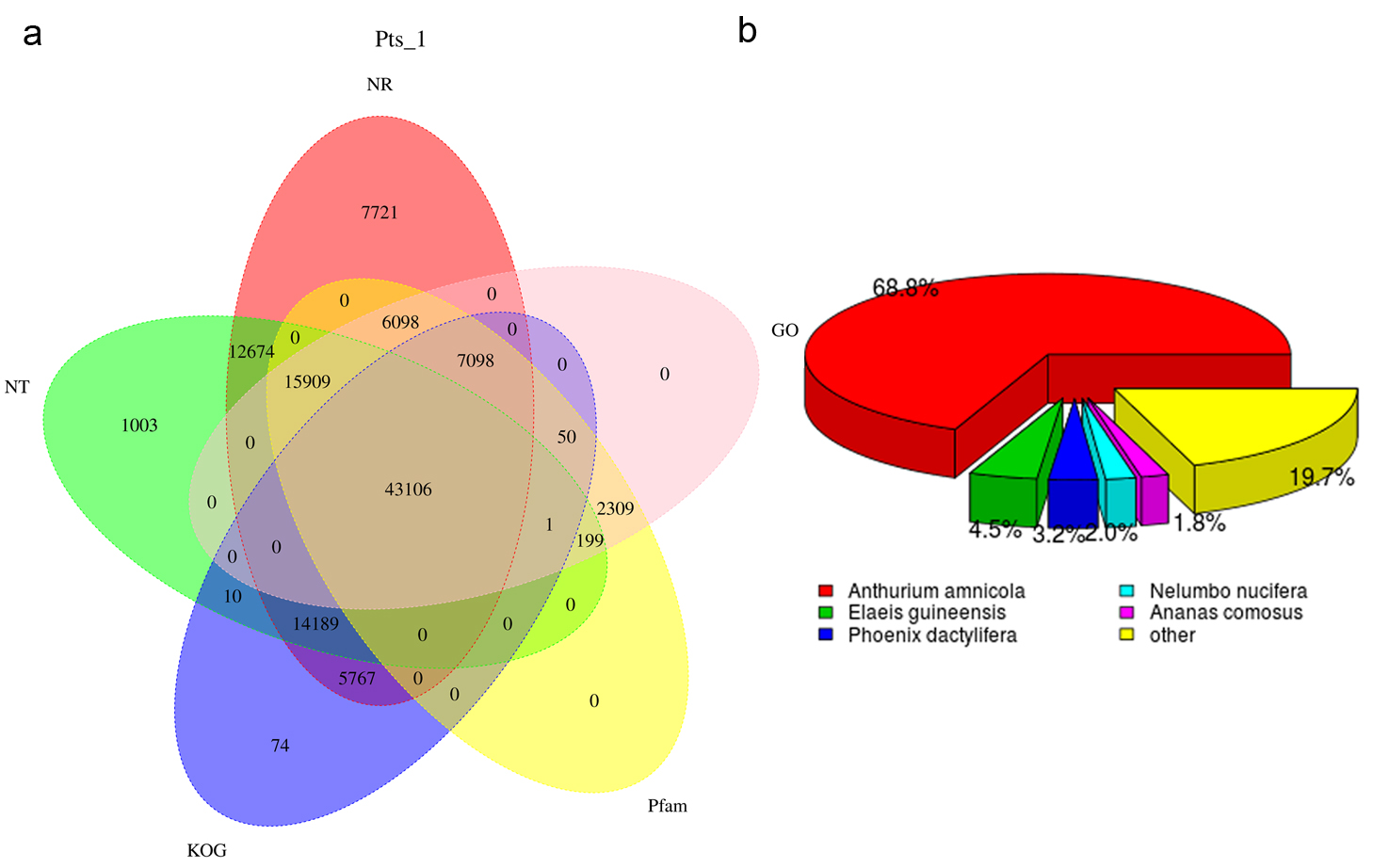

Supplement: Supplementary file 3 — Additional file 3: Fig. S1 Functional annotation and categorization of P. ternata transcripts. a Venn diagram of NR, NT, GO, KOG and Pfam results for the P. ternata transcripts. b Homologous species of P. ternata transcripts. [file 12870_2019_2197_MOESM3_ESM.jpg]

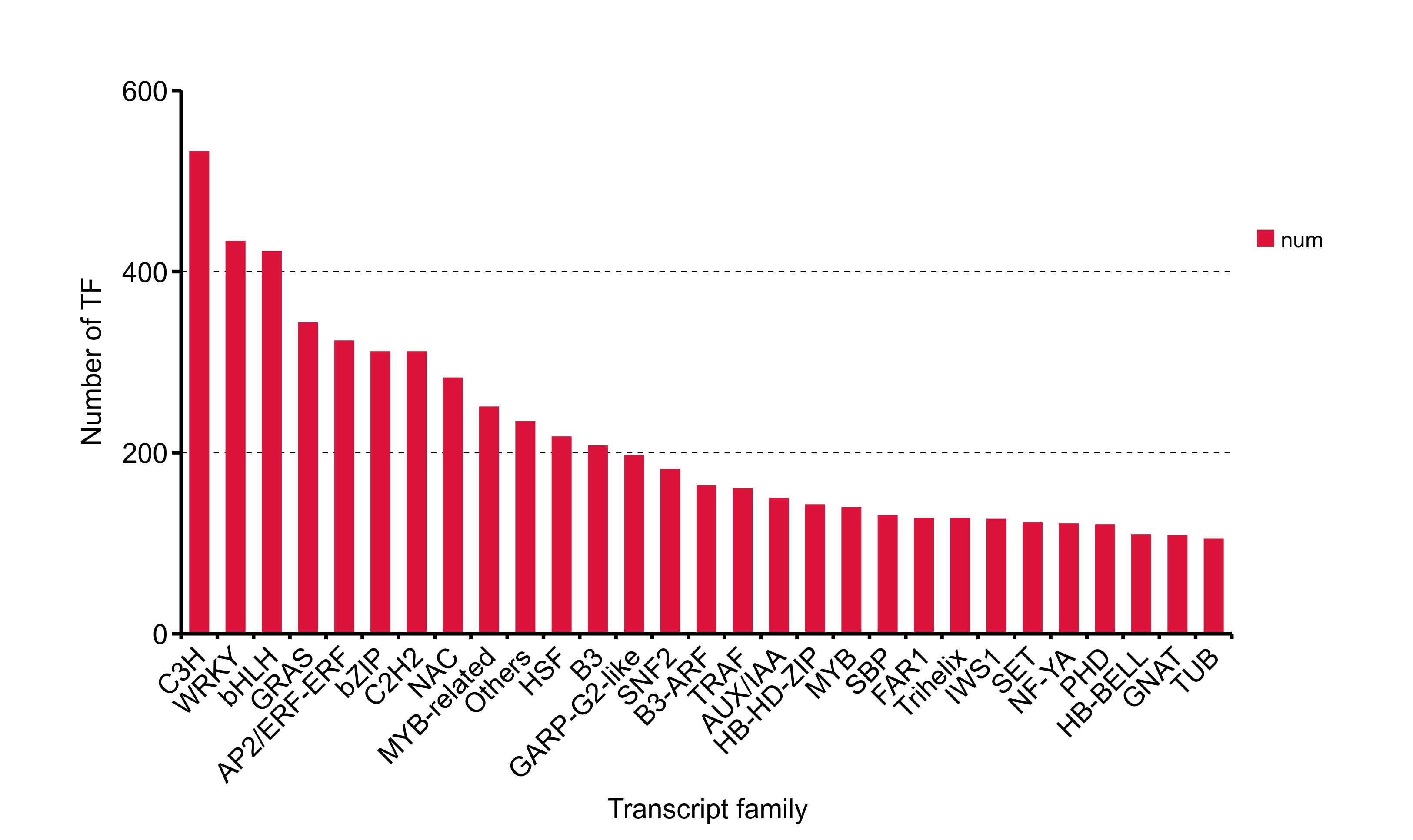

Supplement: Supplementary file 4 — Additional file 4: Fig. S2 Number and family of top 29 TFs predicted by SMRT. [file 12870_2019_2197_MOESM4_ESM.png]

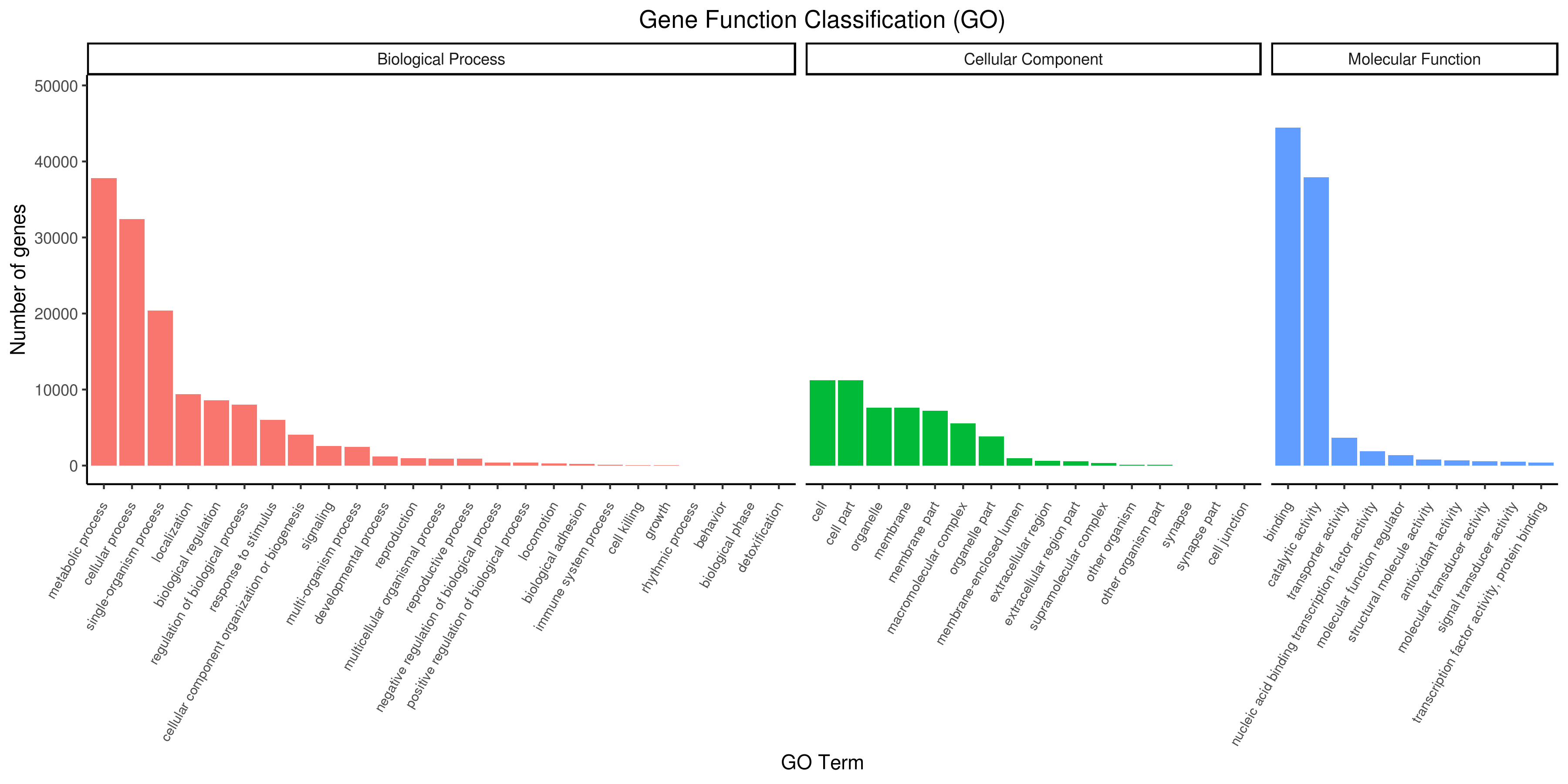

Supplement: Supplementary file 5 — Additional file 5: Fig. S3 Distribution of GO terms for all annotated transcripts in biological process, cellular component and molecular function. [file 12870_2019_2197_MOESM5_ESM.png]

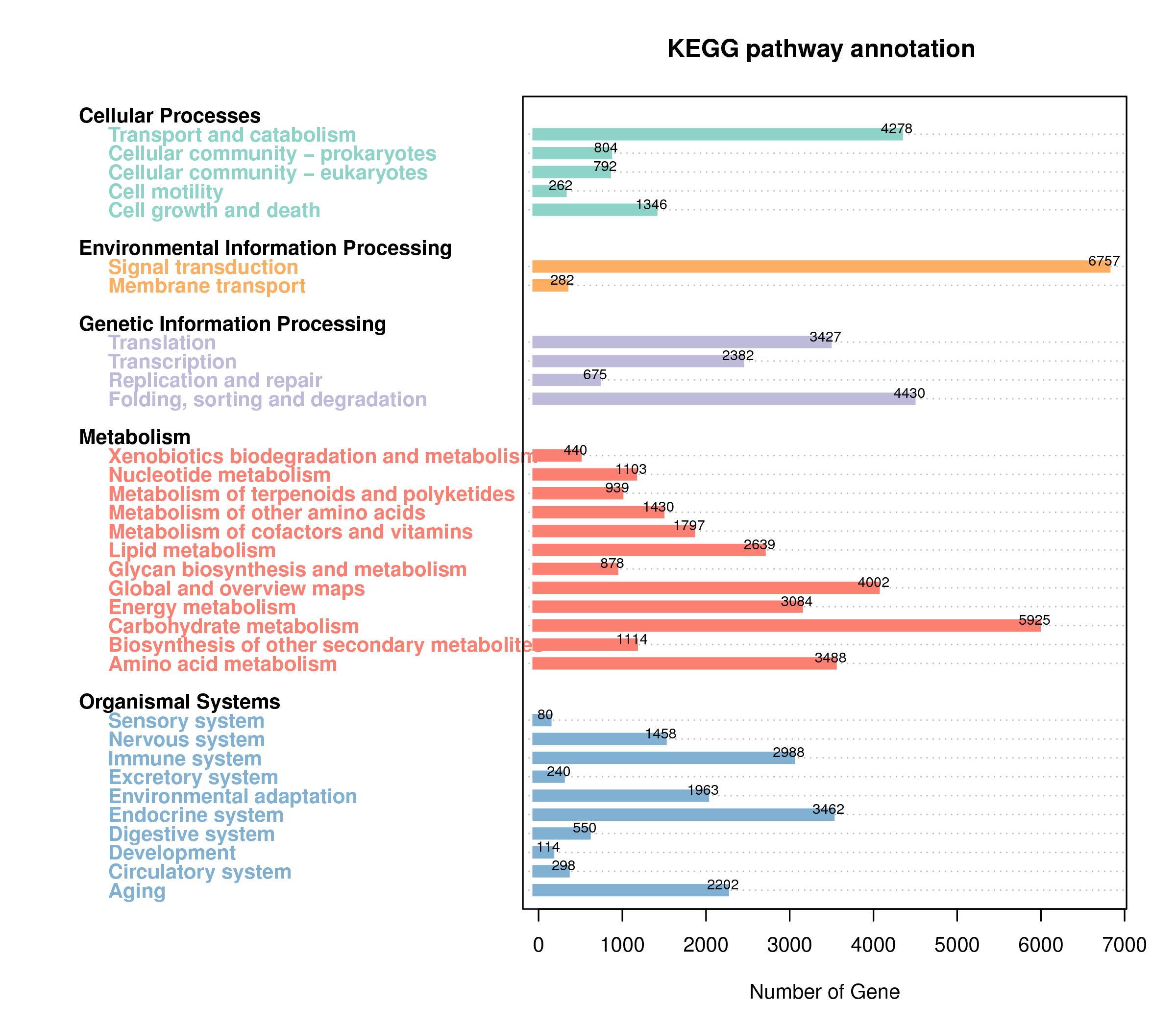

Supplement: Supplementary file 6 — Additional file 6: Fig. S4. KEGG pathways enriched of transcripts. [file 12870_2019_2197_MOESM6_ESM.png]

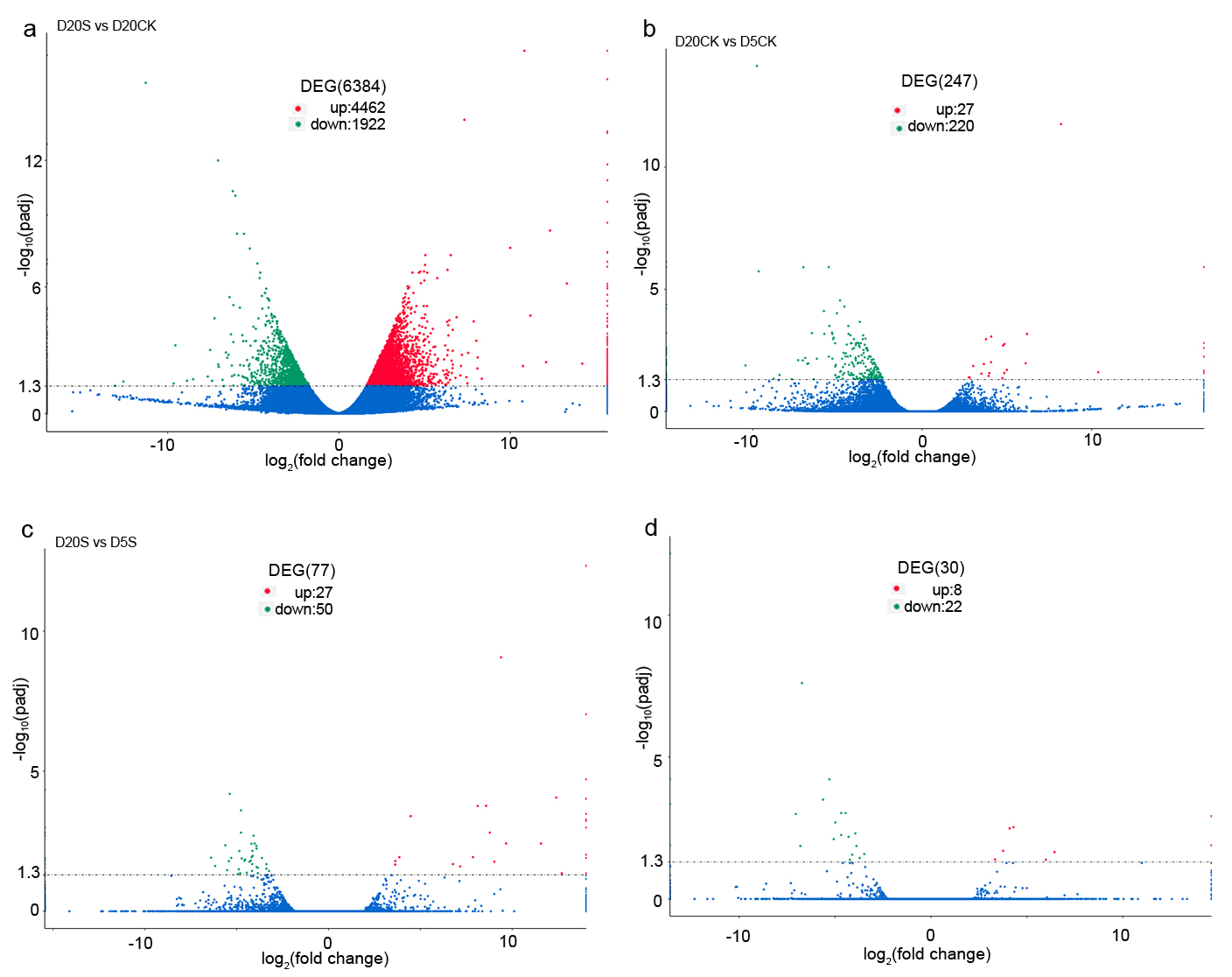

Supplement: Supplementary file 7 — Additional file 7: Fig. S5. Volcano map-analysis of differential expression genes. [file 12870_2019_2197_MOESM7_ESM.jpg]
