# Supplementary material for: Full-length transcriptome analysis of shade-induced promotion of tuber production in Pinellia ternata
Source: BMC Plant Biol. 2019 Dec 18;19:565. doi: 10.1186/s12870-019-2197-9 (PMC6921527; doi:10.1186/s12870-019-2197-9)
Supplement: Supplementary file 8 — Additional file 8: Table S3. Primers used for real-time quantitative PCR. [file 12870_2019_2197_MOESM8_ESM.doc]

**Additional file 8: Table S3.** Primers used for real-time quantitative PCR

| Gene name | Sequence |
| --- | --- |
| *PtPF3*-qPCR-F | ATCTAACACCACCACCTTC |
| *PtPF3*-qPCR-R | GTAGTAGTGCTGCTGCTC |
| *PtHY5*-qPCR-F | GCAGAAGGCAGATAAGGAACAGA |
| *PtHY5*-qPCR-R | ATACACAGCAGTGGAGAGCATTC |
| *PtCAB151*-qPCR-F | AAGGTGAAGGAGATCAAGAA |
| *PtCAB151*-qPCR-R | GAACAGGTTCTCGATGGG |
| *PtRUB*-qPCR-F | AAGCAGGTGGTGTTCTAC |
| *PtRUB*-qPCR-R | AAGCAGGTGGTGTTCTAC |
| *PtCHS*-qPCR-F | GCTCAAAGAGTACGGCAACAT |
| *PtCHS*-qPCR-R | TTCCTTCCTCCTCCGACTTC |
| *PtPOD*-qPCR-F | TGGACAACAACTACTATCG |
| *PtPOD*-qPCR-R | GATGAAGTGGTGGAAGAA |
| *PtSOD*-qPCR-F | CCTATCAGCCATCATCATC |
| *PtSOD*-qPCR-R | CATACAGGCAAGGAAGTC |
| *PtCAT*-qPCR-F | ATGCGGAGAGATACCCTA |
| *PtCAT*-qPCR-R | TGCTTGAAATCGTTCGTTT |
| *PtGR*-qPCR-F | CTATTTGGGCTGTGGGAGATGTC |
| *PtGR*-qPCR-R | CTATTTGGGCTGTGGGAGATGTC |
| *PtARF1*-qPCR-F | CCTCACATTCCATTCCAAG |
| *PtARF1*-qPCR-R | ATAATCTACAGCCACTTCCT |
| *PtARF19*-qPCR-F | TCACTTCTTCAGCAGCAT |
| *PtARF19*-qPCR-R | ATCAAGCATCTCAGTTGGA |
| *PtPIN1*-qPCR-F | GTGATGACGAGGCTGATA |
| *PtPIN1*-qPCR-R | GAGATGGACTTGGCGATA |
| *PtPIN6*-qPCR-F | CTCTTCTTCTCCCGTGTT |
| *PtPIN6*-qPCR-R | CCTATAAGTGAGCCCGATAT |
| *PtSuSy*-qPCR-F | CTCCTCCAAGATGTTCCA |
| *PtSuSy*-qPCR-R | GCTGCGTATTCTGTTGTT |
| *PtXET*-qPCR-F | ACCTACTCCTTCTGCTAC |
| *PtXET*-qPCR-R | GTCGTTGAACTTGTCCTT |
| *PtRGP*-qPCR-F | TGGCAGGAAGACATCATT |
| *PtRGP*-qPCR-R | CTTGACGAGATTGCTCAG |
| *Pt18S*-qPCR-F | CGCATATAAATAAACGGAGGAA |
| *Pt18S*-qPCR-R | GACGCTTCTACAGACTACA |
